# Dosimetric characterization of a new surface‐conforming electron MLC prototype

**DOI:** 10.1002/acm2.14173

**Published:** 2023-10-19

**Authors:** Holly M. (Parenica) Paschal, Christopher N. Kabat, Thomas Martin, Daniel Saenz, Pamela Myers, Karl Rasmussen, Sotirios Stathakis, Mark Bonnen, Nikos Papanikolaou, Neil Kirby

**Affiliations:** ^1^ Department of Radiation Oncology, School of Medicine The University of Texas Health Science Center at San Antonio San Antonio Texas USA

**Keywords:** electron therapy, multi‐leaf collimator, skin collimation

## Abstract

The purpose is to reduce normal tissue radiation toxicity for electron therapy through the creation of a surface‐conforming electron multileaf collimator (SCEM). The SCEM combines the benefits of skin collimation, electron conformal radiotherapy, and modulated electron radiotherapy.

An early concept for the SCEM was constructed. It consists of leaves that protrude towards the patient, allowing the leaves to conform closely to irregular patient surfaces. The leaves are made of acrylic to decrease bremsstrahlung, thereby decreasing the out‐of‐field dose. Water tank scans were performed with the SCEM in place for various field sizes for all available electron energies (6, 9, 12, and 15 MeV) with a 0.5 cm air gap to the water surface at 100 cm source‐to‐surface distance (SSD). These measurements were compared with Cerrobend cutouts with the field size‐matched at 100 and 110 cm SSD. Output factor measurements were taken in solid water for each energy at d_max_ for both the cerrobend cutouts and SCEM at 100 cm SSD.

Percent depth dose (PDD) curves for the SCEM shifted shallower for all energies and field sizes. The SCEM also produced a higher surface dose relative to Cerrobend cutouts, with the maximum being a 9.8% increase for the 3 cm × 9 cm field at 9 MeV. When compared to the Cerrobend cutouts at 110 cm SSD, the SCEM showed a significant decrease in the penumbra, particularly for lower energies (i.e., 6 and 9 MeV). The SCEM also showed reduced out‐of‐field dose and lower bremsstrahlung production than the Cerrobend cutouts. The SCEM provides significant improvement in the penumbra and out‐of‐field dose by allowing collimation close to the skin surface compared to Cerrobend cutouts. However, the added scatter from the SCEM increases shallow PDD values. Future work will focus on reducing this scatter while maintaining the penumbra and out‐of‐field benefits the SCEM has over conventional collimation.

## 1 INTRODUCTION

Radiation therapy is indicated for more than half of all cancer patient treatments.^1^ Therapeutic electron beams, typically ranging from 6 to 20 MeV, are ideal for treating superficial tumors due to their limited range in tissue and sharper dose fall‐off in comparison to photon beams. Despite this advantage, it is commonly only used for treating lumpectomy cavities and cancers affecting the skin. While electrons primarily interact with tissues (which leads to a larger penumbra), electrons are also easily scattered in air which can lead to tissues outside of the field receiving dose. To combat this issue, the electron beam must be collimated close to the patient's surface using applicators or cones. However, electron applicators do not conform to the surface and leave non‐negligible air gaps, allowing electrons to scatter and deposit dose outside of the intended target. Furthermore, patient‐specific collimation inserts, or “cutouts”, are often made from a low‐temperature casting metal, Cerrobend. Cerrobend is made from toxic metals such as lead and is classified as a level‐4 health hazard (extreme danger) by the National Fire Protection Association. Cerrobend is carcinogenic and prolonged exposure can cause irreversible damage to the kidneys, liver, skeletal structures, and central nervous system.

Skin collimation is a technique that is sometimes used to reduce scatter dose from electrons. Lead is placed directly on the patient's surface, significantly reducing the out‐of‐field dose.^2^ In addition to surface‐blocking techniques, bolus is used to improve the utility of electron therapy. For this, an amount of material is placed on the patient surface to modify the electron range. In its simplest implementation, a thin slab of material will be placed over a treatment area and used to alter the range to a desirable stopping position. Electron conformal radiotherapy (ECRT) is a more advanced application of bolus. For this, three‐dimensional printed boluses are custom‐tailored for a patient to conform the electron radiation to the distal tumor boundary. There will be minimal bolus in locations where the tumor is the deepest and conversely thick bolus will overlay shallow tumor locations. ECRT enables electrons to treat a variety of other sites: post‐mastectomy chest wall, head and neck, and paraspinal muscles.^3^, ^4^, ^5^, ^6^, ^7^ One complication with this technique is that the thick areas of this bolus will have a similar effect as a large air gap. It will blur out the edge of an electron beam. Thus, lateral dose fall‐off is sacrificed for range modulation ability.

Modulated electron therapy (MERT) is another technique that has been applied to improve the utility of electrons. In this technique, multileaf collimators (MLCs) are used to shape the electron fluence. In contrast to ECRT, range modulation is achieved by mixing electron energies. One technique for this is to position a patient close to a linear accelerator head and collimate with the photon MLCs.^8^, ^9^, ^10^, ^11^ Another approach has been the use of add‐on MLCs.^12^, ^13^, ^14^, ^15^ Due to patient collision issues, the collimating material of these previous MLC‐based techniques must be further away from the patient surface than conventional applicators. This placement issue fundamentally increases the amount of radiation scattered to normal tissue and limits the ability of these techniques to produce sharp dose falloffs. However, even with these limitations, MERT has still shown the potential to treat breast, chest wall, and scalp tumors while sparing adjacent healthy tissue better than photon therapy.^16^, ^17^, ^18^ Eldib et al. studied partial scalp radiotherapy and found that MERT reduced the mean brain dose by 59% compared to photon IMRT treatments.^18^ Ma et al. demonstrated a reduction in the maximum heart and lung dose by 20 Gy for MERT compared to photon breast treatments.^17^ Gauer et al. displayed a 35% (2.2 Gy) reduction in the mean heart dose with MERT compared to whole breast conventional photon therapy.^16^ Major coronary events are found to increase linearly (with no threshold) with the mean heart dose at a rate of 7.4% per Gy.^19^ Thus, it is anticipated that this 2.2 Gy reduction in mean heart dose would lower major coronary events for these patients by 16.3%. Given that there are over two million breast cancer cases per year worldwide, this reduction can have a profound global health impact.^20^

It is critical to control radiation delivered not only to tumors and adjacent structures but also the low levels that are scattered elsewhere in the body. Low levels of radiation exposure increase the risk of secondary malignancies. The Women's Environmental, Cancer, and Radiation Epidemiology (WECARE) study evaluated the risk of developing secondary cancer from breast radiotherapy.^21^ They found that women less than 40 years old, who were exposed to more than 1 Gy of radiation to the contralateral breast, had a 2.5‐fold increase in risk compared to unexposed women. This dose is only 2% of a common breast irradiation prescription, 50 Gy. For this reason, a radiotherapy strategy that reduces these low levels of scattered radiation has the potential to significantly impact the risk for radiation‐induced malignancies.

**FIGURE 1:**
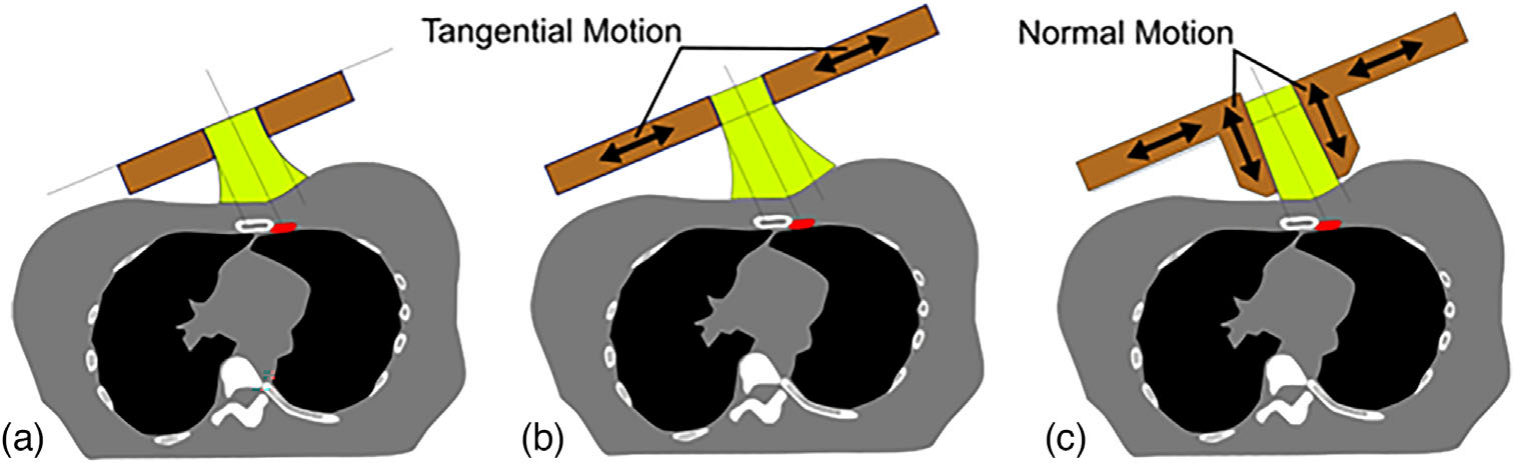
Comparison of MLC positioning and leaf motion relative to the patient surface for stationary leaves (a), leaves moving tangentially (b), and leaves moving normally and tangentially (c). MLC, multileaf collimator.

Ultimately, the best way to reduce this toxicity is by lowering the radiation dose received by normal tissue. The ideal dose distribution should have a sharp falloff in the penumbra to spare lateral tissue, modulate its range to spare tissue beyond the target and minimize dose in the tail of the distribution to reduce the potential for secondary malignancies. Skin collimation, ECRT, and MERT techniques have a variety of strengths, but none possess all these ideal characteristics. Our approach to reducing normal tissue toxicity is to engineer an MLC system that conforms to the patient surface. This surface‐conforming electron MLC (SCEM) should produce large reductions in normal tissue doses found with MERT, while also minimizing low levels of radiation exposure throughout the body. In the context of breast radiotherapy, this would reduce the risk of inducing both major coronary events and secondary malignancies. In addition to dosimetric advantages, an MLC system would avoid patient‐specific fabrication and handling of Cerrobend. The design goal for the SCEM device is for leaves to protrude toward the patient and move not only tangential to the patient surface (as other MLCs do) but also normal to the patient surface. Figure 1 displays a comparison of conventional collimation and other MLC approaches to the SCEM strategy. The two‐dimensional motion allows the MLCs to be positioned close to even irregular patient surfaces. This dramatically improves how sharply the dose falls off outside of a target and enables better radiation sparing of adjacent normal tissue. Also, these MLCs are made of plastic, which produces less bremsstrahlung than metal collimation. We have constructed a SCEM prototype for evaluation of the concept. It has leaves that protrude toward the surface (as in Figure 1c); however, the prototype does not have MLC motion normal to the surface. Instead, the dosimetric advantages are investigated with flat phantom geometries.

## 2 METHODS AND MATERIALS

### 2.1 Design of SCEM

**FIGURE 2:**
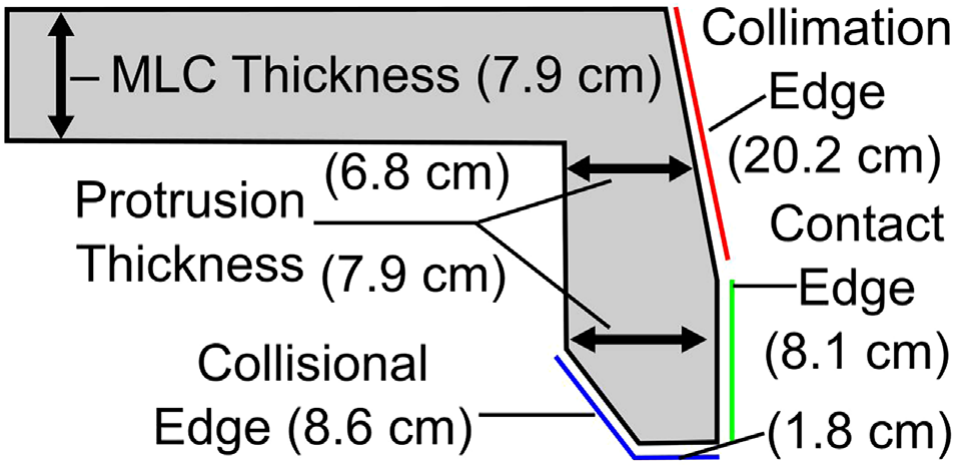
Design of the SCEM for a single leaf. SCEM, surface‐conforming electron multileaf collimator.

MLC leaves were cut into the simple shape shown in Figure 2. Electrons with an energy of 15 MeV have a range of 6.4 cm in acrylic.^22^ The MLC thickness and contact edge were made to be 8 cm to minimize intra‐ and inter‐MLC transmission (when opposing MLCs touch). The collimation edge was tilted relative to the contact edge to avoid unnecessary clipping of electrons on a trajectory to the treatment site. The protrusion thickness was 8 cm near the contact edge but tapered down to 7 cm at its smallest (due to this tilt). The outside corner of the MLC protrusion was cut to make the collisional edge. This would enable the MLC to be as close as possible to an irregular patient surface.

**FIGURE 3:**
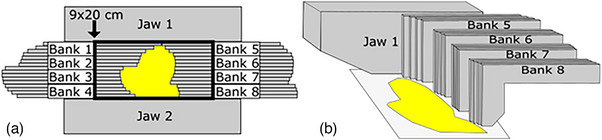
A beam's eye view (a) and sideview (b) of the SCEM in a bank with the jaws. The width of the bank of leaves is fixed a 9 cm in the in‐plane direction and the leaves can open to a maximum of 20 cm in the cross‐plane direction.

**FIGURE 4:**
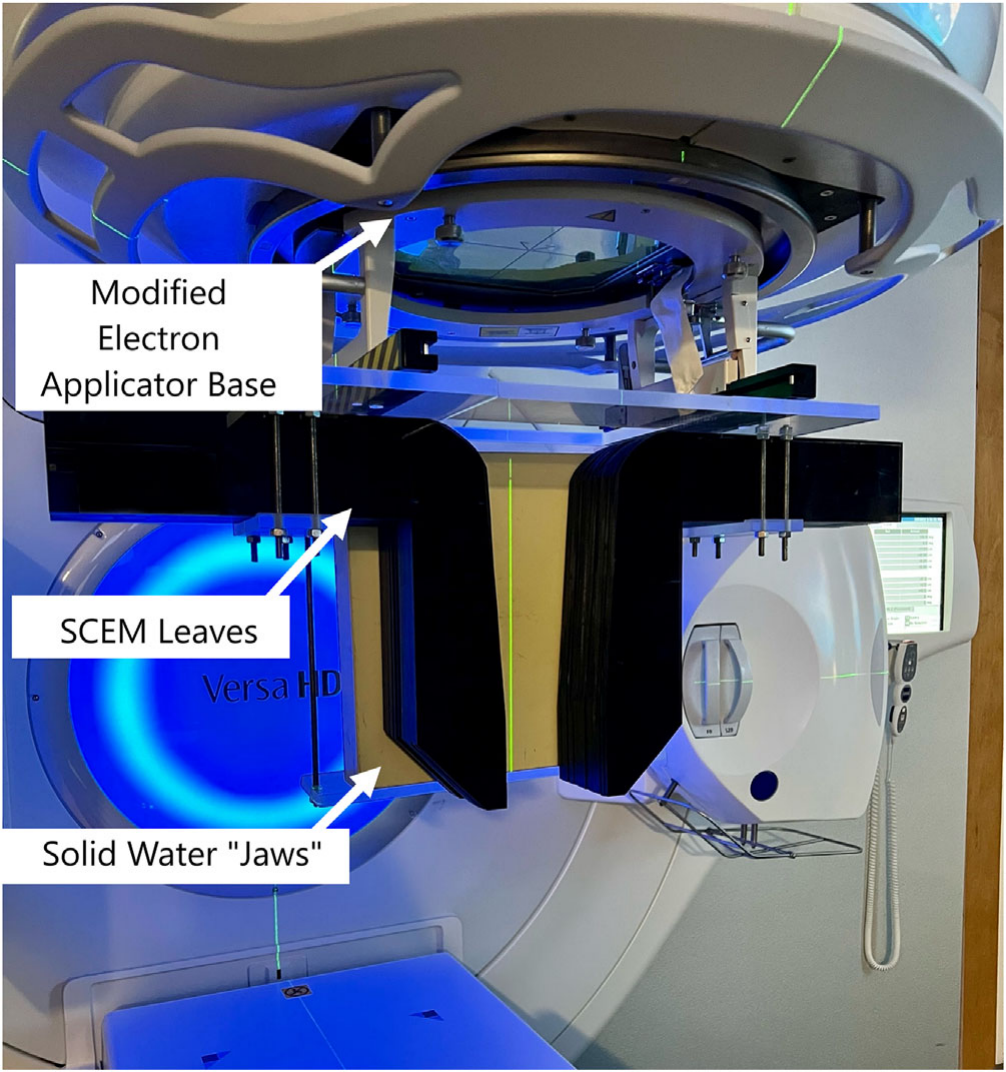
The SCEM attached to the linac with one solid water “jaw” removed.

A total of 32 MLC leaves were made, with 16 being put into each opposing bank, as seen in Figure 3. The MLCs were all 5.8 mm in thickness, meaning that 16 leaves together were approximately 9 cm thick. The long‐term vision for this device would be to group MLCs into smaller banks (Bank 1−8 in Figure 3), where each would be able to move normally to the surface independently of the others. In the current prototype though, this motion normal to the surface is not possible. An MLC support structure was fabricated on a block tray fixture (see Figure 4). The MLCs were fixed to an acrylic sheet that was bolted to the block tray. Additionally, a collimator head dongle was wired for the Versa HD to interpret the attachment as a 20 cm × 20 cm electron cone. This support structure allows for the MLCs to be pulled back as far as 10 cm from the central axis and as far forward as 5 cm beyond it. This allowed the MLC to make field sizes as large as 9 cm × 20 cm. The long‐term vision for this device would also use jaws to terminate the MLC field at each end (see Figure 3). In the current prototype, 30 cm × 30 cm × 5 cm solid water slabs are used as these jaws. Figure 4 displays the SCEM attached to the head of the linac with one solid water jaw removed to better visualize the leaves.

### 2.2 Measurements

Water tank scans were taken using the PTW BEAMSCAN (PTW, Freiburg) with the PTW 60012 Diode. Cerrobend cutouts were created for three field sizes (3 cm × 9 cm, 5 cm × 9 cm, and 10 cm × 9 cm) that were defined at the surface. Measurements for the cerrobend cutouts were taken at 100 cm SSD and 110 cm SSD (a more clinically relevant SSD) for all available energies on the Elekta Versa HD linear accelerator (6, 9, 12, and 15 MeV). Percent depth dose (PDD) curves were measured for each energy and field size and included depths several centimeters beyond the practical range (R_p_) to better characterize the bremsstrahlung tails. Cross‐plane profiles were taken at a variety of depths ranging from near‐surface (i.e., 1 mm) to depths beyond the practical range. Additionally, cross‐plane profiles were scanned several centimeters beyond the field edges to characterize out‐of‐field dose.

The SCEM leaves were adjusted to match the field sizes of the cerrobend cutouts at the water surface within 1 mm, and all SCEM measurements were made at 100 cm SSD, creating a 0.5 cm air gap from the tip of the SCEM to the water surface. MLCs were moved manually. Field sizes were validated visually with a ruler and were additionally verified in the PTW BEAMSCAN software from the dose profiles. PDD curves were measured for each energy and field size, and cross‐plane profiles were taken at the same depths as the cerrobend cutouts. Output factor measurements were taken in solid water using a PTW Semiflex ion chamber (0.125 cm^3^) for each energy at d_max_ for both the cerrobend cutouts and SCEM, both at 100 cm SSD. Additionally, output factors were taken with a fixed phantom size using a MiniPhantom (Standard Imaging, Middleton, WI). The MiniPhantom was oriented horizontally and was shifted for each energy so that the chamber center would be at the same distance from the source as in the solid water measurements. This allowed for an evaluation of output changes without increased lateral scatter from the phantom material. Profile data was exported to Matlab to generate isodose lines for comparing the SCEM with the Cerrobend cutouts. PDD and output factor data were exported to Excel to generate graphs for the respective data sets.

## 3 RESULTS

**FIGURE 5:**
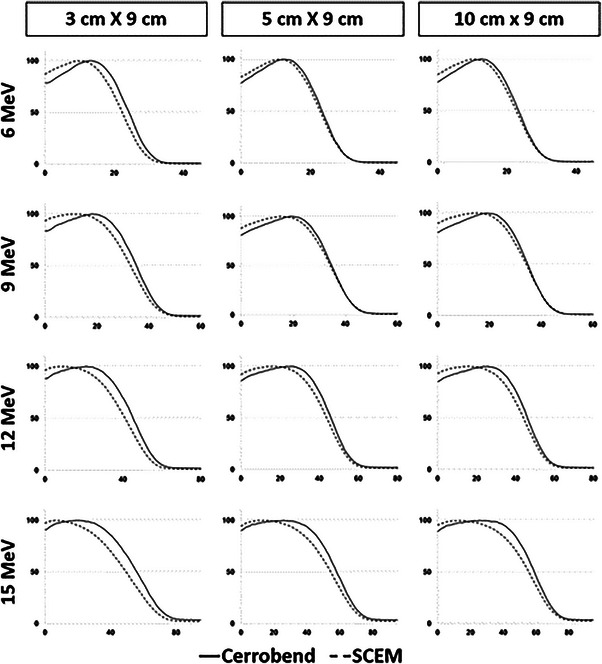
PDD curves for the SCEM (dashed) and Cerrobend cutouts (solid). The *y*‐axis is the central‐axis percent depth dose relative to the maximum and the *x*‐axis is the central axis depth in millimeters. PDD, percent depth dose.

The PDD curves for the SCEM and cerrobend cutouts can be seen in Figure 5. The PDDs displayed for the cerrobend cutouts were taken at 100 cm SSD, as there was no significant difference from the PDDs for cerrobend cutouts at 110 cm SSD. Table 1 displays the R50 values for the SCEM and Cerrobend cutouts at 100 cm SSD for all energies and field sizes. Table 2 summarizes the surface dose (relative to the maximum dose) and the bremsstrahlung tail (i.e., the central axis dose relative to d_max_ 1 cm beyond the practical range) for the SCEM and the Cerrobend cutout at 100 cm SSD.

**TABLE 1:** R50 values for the SCEM and Cerrobend cutouts at 100 cm SSD for all energies and fields sizes.

		6 MeV	9 MeV	12 MeV	15 MeV
3 cm × 9 cm	Cerrobend	24.31 mm	35.26 mm	45.33 mm	56.02 mm
	SCEM	22.37 mm	33.05 mm	41.21 mm	49.55 mm
5 cm × 9 cm	Cerrobend	22.35 mm	34.84 mm	46.07 mm	58.09 mm
	SCEM	22.79 mm	34.26 mm	43.89 mm	54.53 mm
10 cm × 9 cm	Cerrobend	23.41 mm	34.86 mm	46.14 mm	59.01 mm
	SCEM	22.73 mm	34.23 mm	44.14 mm	55.62 mm

Abbreviations: SCEM, surface‐conforming electron multileaf collimator; SSD, source‐to‐surface distance.

**TABLE 2:** Surface dose (% relative to d_max_) and the bremsstrahlung tail (i.e., the central axis dose relative to d_max_ 1 cm beyond the practical range) for the Cerrobend cutout at 100 cm SSD and the SCEM.

		Surface dose		Bremsstrahlung tail	
		Cerrobend	SCEM	Cerrobend	SCEM
3 cm × 9 cm	6 MeV	78.7%	87.2%	1.2%	0.8%
	9 MeV	83.7%	93.5%	1.7%	1.3%
	12 MeV	88.4%	96.4%	2.4%	1.5%
	15 MeV	91.0%	97.3%	3.9%	2.4%
5 cm × 9 cm	6 MeV	77.0%	83.1%	1.2%	1.0%
	9 MeV	80.9%	88.0%	1.6%	1.4%
	12 MeV	85.7%	92.2%	2.7%	1.7%
	15 MeV	89.9%	94.5%	3.9%	2.6%
10 cm × 9 cm	6 MeV	77.9%	85.2%	1.2%	1.1%
	9 MeV	80.9%	90.1%	1.6%	1.5%
	12 MeV	85.1%	93.1%	2.5%	1.8%
	15 MeV	89.3%	95.3%	3.8%	2.8%

Abbreviations: SCEM, surface‐conforming electron multileaf collimator; SSD, source‐to‐surface distance.

**FIGURE 6:**
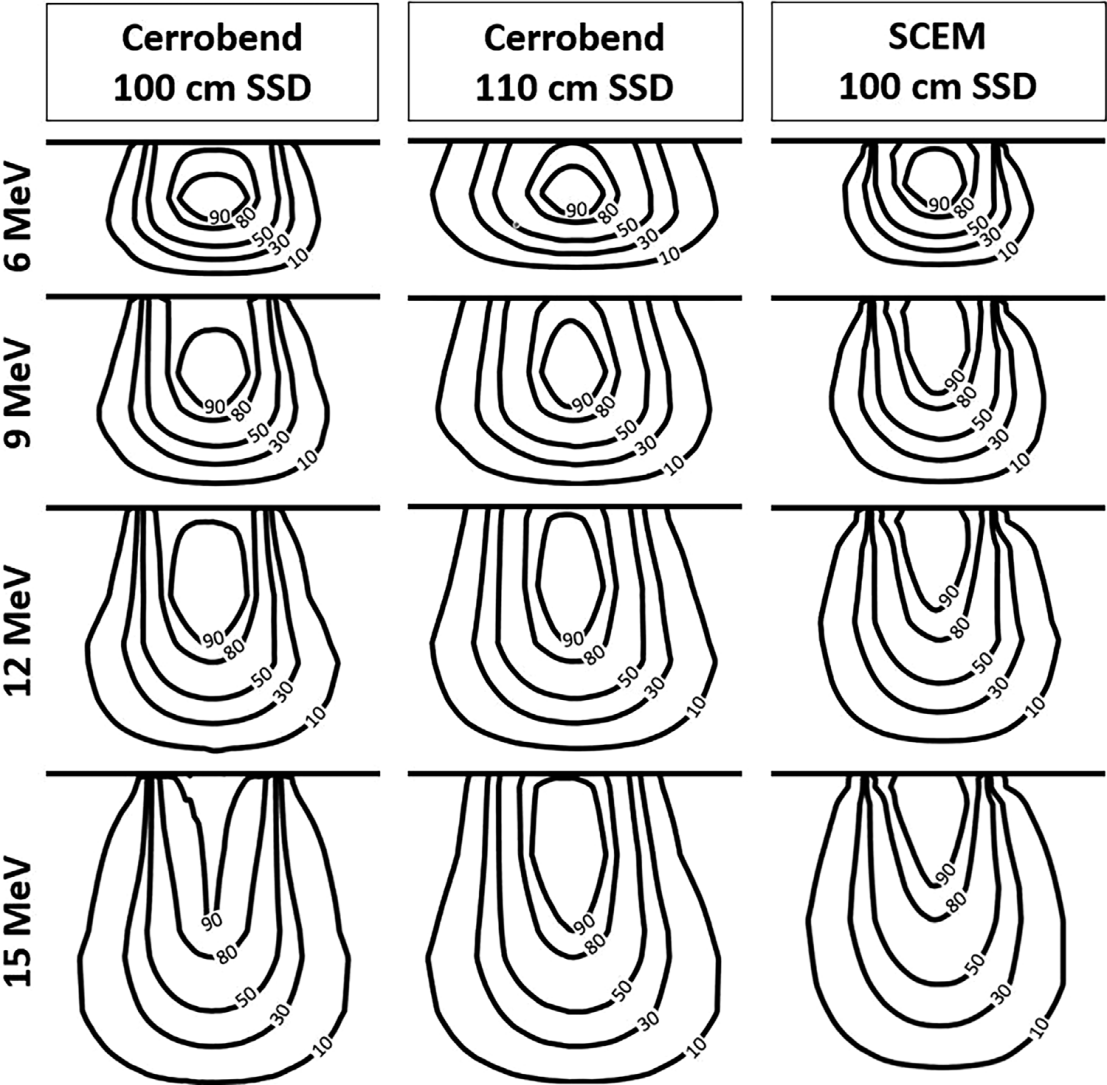
A comparison of the isodose lines for the cerrobend cutout at 100 cm SSD, cerrobend cutout at 110 cm SSD, and the SCEM for the 3 cm × 9 cm field size for each energy. The isodose lines shown from lowest to highest are 10%, 30%, 50%, 80%, and 90%. SCEM, surface‐conforming electron multileaf collimator; SSD, source‐to‐surface distance.

**FIGURE 7:**
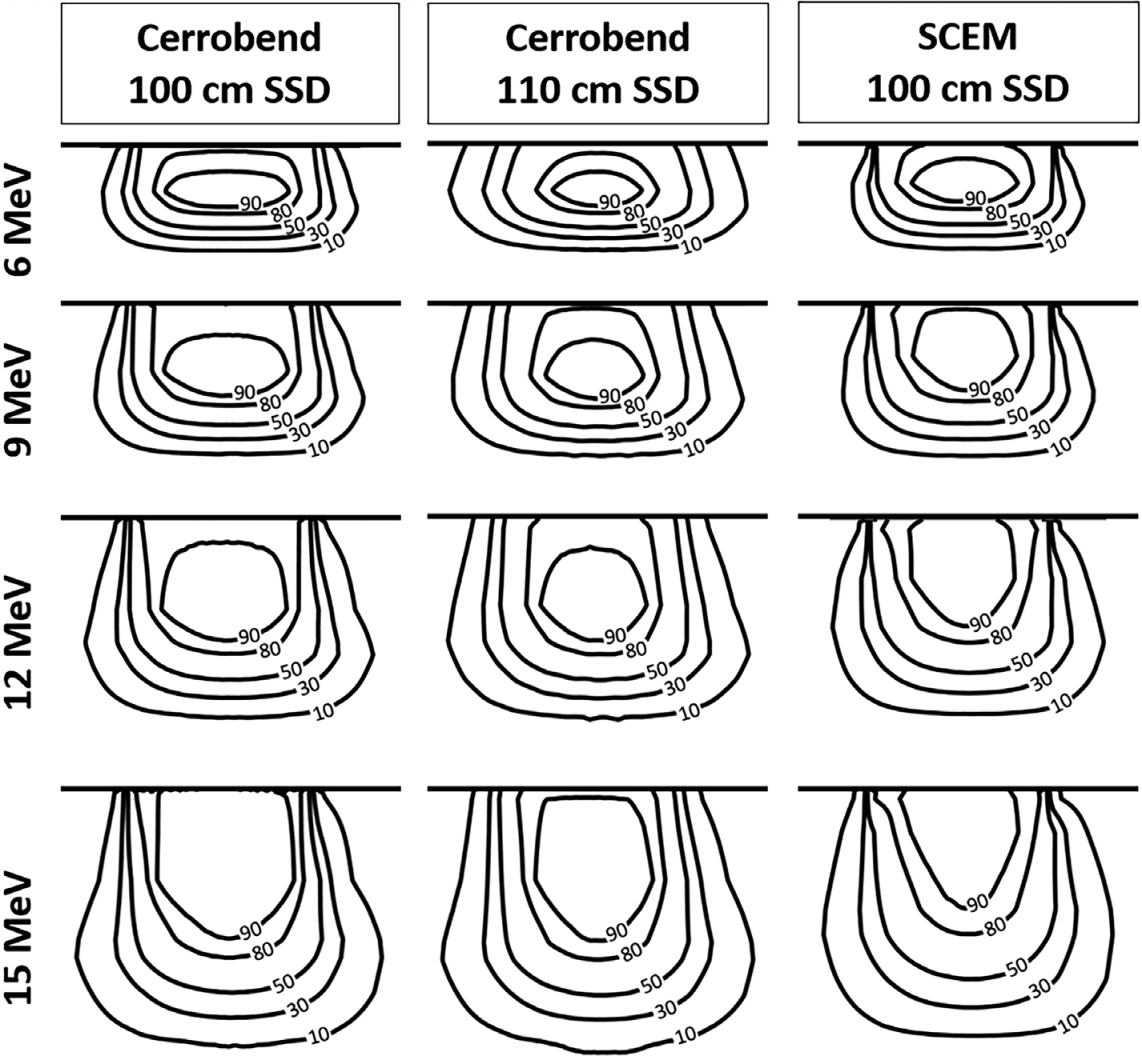
A comparison of the isodose lines for the cerrobend cutout at 100 cm SSD, cerrobend cutout at 110 cm SSD, and the SCEM for the 5 cm × 9 cm field size for each energy. The isodose lines shown from lowest to highest are 10%, 30%, 50%, 80%, and 90%. SCEM, surface‐conforming electron multileaf collimator; SSD, source‐to‐surface distance.

**FIGURE 8:**
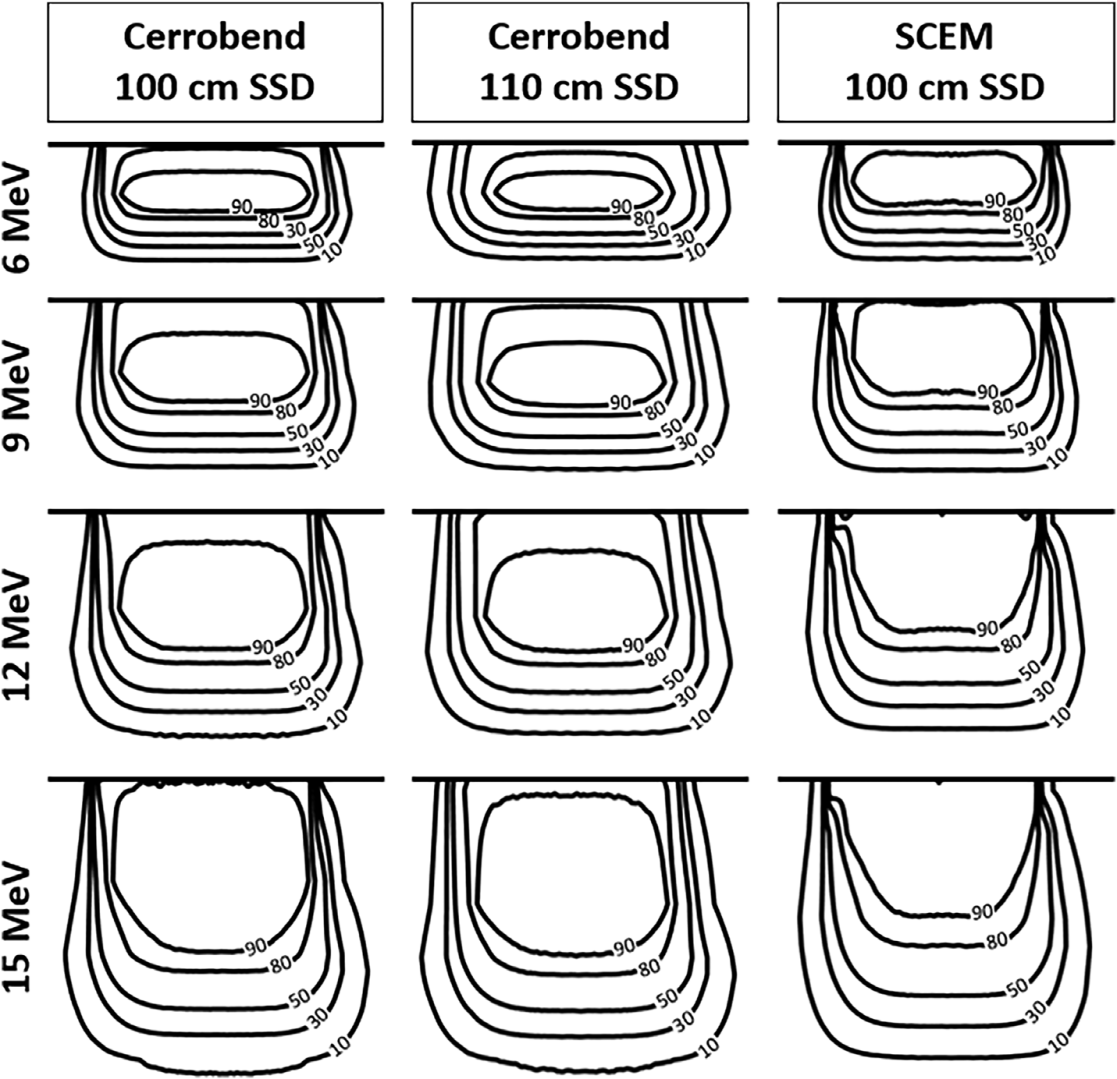
A comparison of the isodose lines for the cerrobend cutout at 100 cm SSD, cerrobend cutout at 110 cm SSD, and the SCEM for the 10 cm × 9 cm field size for each energy. The isodose lines shown from lowest to highest are 10%, 30%, 50%, 80%, and 90%. SCEM, surface‐conforming electron multileaf collimator; SSD, source‐to‐surface distance.

A comparison of the isodose lines for the cerrobend cutout at 100 cm SSD, cerrobend cutout at 110 cm SSD, and the SCEM for the 3 cm × 9 cm, 5 cm × 9 cm, and 10 cm × 9 cm field sizes can be seen in Figures 6, 7, 8, respectively. Table 3 displays the penumbra, which is defined as the lateral distance between the 80% and 20% isodose lines at d_max_. A comparison of the dose transmission 3 cm from the field edge for the SCEM, and cerrobend cutouts (100 cm SSD and 110 cm SSD) for all energies and field sizes can be seen in Table 4.

**TABLE 3:** Comparison of penumbra size (defined as the distance between 20% and 80% of central axis dose) at d_max_ for each field size and energy.

		6 MeV	9 MeV	12 MeV	15 MeV
3 cm × 9 cm	SCEM	8.7 mm	9.6 mm	9.3 mm	7.7 mm
	Cerrobend (100 cm SSD)	9.8 mm	9.7 mm	8.7 mm	6.8 mm
	Cerrobend (110 cm SSD)	16.5 mm	14.7 mm	12.9 mm	10.6 mm
5 cm × 9 cm	SCEM	9.6 mm	11.2 mm	12.1 mm	10.7 mm
	Cerrobend (100 cm SSD)	10.9 mm	11.4 mm	11.6 mm	9.6 mm
	Cerrobend (110 cm SSD)	18.6 mm	16.5 mm	15.2 mm	12.7 mm
	SCEM	7.8 mm	9.3 mm	10.7 mm	9.6 mm
10 cm × 9 cm	Cerrobend (100 cm SSD)	10.3 mm	10.8 mm	11.3 mm	9.6 mm
	Cerrobend (110 cm SSD)	18.9 mm	16.4 mm	15.3 mm	12.8 mm

Abbreviations: SCEM, surface‐conforming electron multileaf collimator; SSD, source‐to‐surface distance.

**TABLE 4:** Comparison of dose transmission 3 cm outside the field edge for the SCEM and Cerrobend cutout (100 cm SSD and 110 cm SSD) at d_max_ for all field sizes and energies.

		6 MeV	9 MeV	12 MeV	15 MeV
3 cm × 9 cm	SCEM	0.2%	0.4%	0.7%	1.2%
	Cerrobend (100 cm SSD)	1.2%	1.7%	2.6%	3.9%
	Cerrobend (110 cm SSD)	2.0%	1.8%	2.2%	3.0%
5 cm × 9 cm	SCEM	0.2%	0.3%	0.7%	1.3%
	Cerrobend (100 cm SSD)	0.9%	1.3%	2.1%	3.2%
	Cerrobend (110 cm SSD)	2.1%	1.9%	2.3%	2.9%
	SCEM	0.2%	0.4%	1.0%	1.7%
10 cm × 9 cm	Cerrobend (100 cm SSD)	0.8%	1.1%	1.7%	2.4%
	Cerrobend (110 cm SSD)	2.6%	2.1%	2.5%	2.8%

Abbreviations: SCEM, surface‐conforming electron multileaf collimator; SSD, source‐to‐surface distance.

**FIGURE 9:**
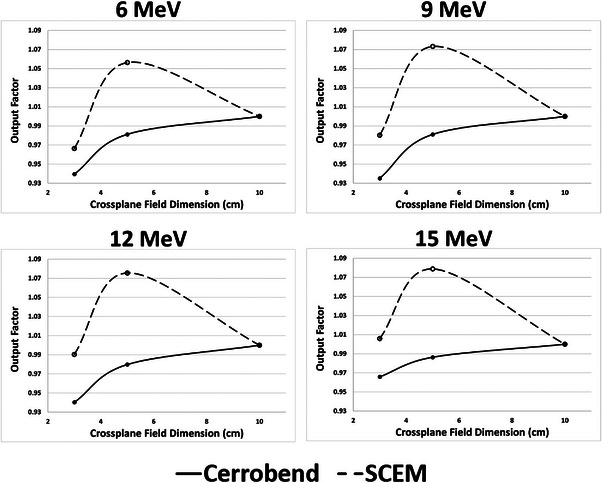
Output factors for each energy for the SCEM (dashed) and Cerrobend cutouts (solid), normalized to the 10 cm × 9 cm field size. The *x*‐axis represents the cross‐plane dimension (i.e., the variable dimension) of the SCEM.

**FIGURE 10:**
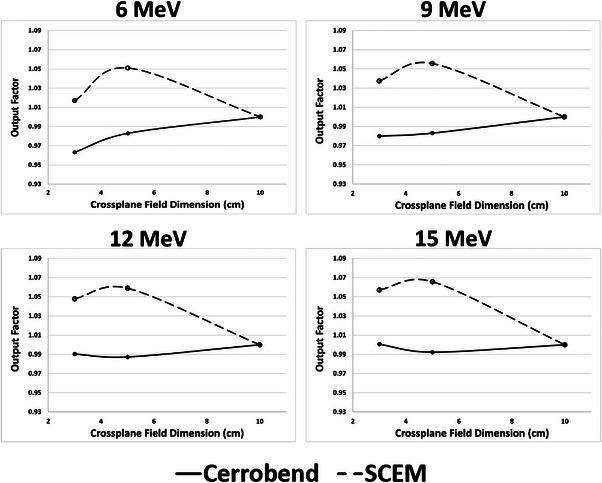
MiniPhantom output factors for each energy for the SCEM (dashed) and Cerrobend cutouts (solid), normalized to the 10 cm × 9 cm field size. The *x*‐axis represents the cross‐plane dimension (i.e., the variable dimension) of the SCEM.

Plots of the output factors taken in solid water (normalized to the 10 cm × 9 cm field) can be seen in Figure 9. The 5 cm × 9 cm field showed the highest relative output and increased with increasing energy. The relative output of the 3 cm × 9 cm field was less than the 10 cm × 9 cm field for all energies except for 15 MeV, where it was slightly greater. Plots of the output factors taken with the MiniPhantom (normalized to the 10 cm × 9 cm field) can be seen in Figure 10. Above 6 MeV, the output for the Cerrobend cutout remains relatively constant, varying less than 2% from the output of the 10 cm × 9 cm field. The SCEM output for the smaller field sizes (3 cm × 9 and 5 cm × 9 cm) varied considerably when compared to the 10 cm × 9 cm field, with this effect becoming more pronounced with increasing energy. The highest relative output was seen for the 5 cm × 9 cm field at 15 MeV, where it was approximately 7% higher than the 10 cm × 9 cm field. It is also worth noting that the raw measured charge readings were much higher for the SCEM than for the Cerrobend cutouts. Table 5 displays the ratios of raw charge readings (SCEM to Cerrobend cutout) for the 10 cm × 9 cm field for all energies for both the solid water and MiniPhantom setups. For both experimental setups, the measured charge readings were at least 23.6% greater for the SCEM than for the Cerrobend cutouts for all energies.

**TABLE 5:** Ratio of raw charge readings (SCEM to Cerrobend cutout) for the 10 cm × 9 cm field for all energies.

	6 MeV	9 MeV	12 MeV	15 MeV
Solid Water	1.258	1.262	1.266	1.291
MiniPhantom	1.236	1.325	1.345	1.360

Abbreviation: SCEM, surface‐conforming electron multileaf collimator.

## 4 DISCUSSION

The goal of this work was to design an electron collimation system that combines the advantages of skin collimation and ECRT and characterize the dosimetric properties of the initial prototype. While this study did not directly utilize MERT, the SCEM has the potential to combine its collimation abilities with energy variation to achieve MERT. The initial SCEM prototype provides an advantage over lead skin collimation in that it eliminates the need to cut lead for individual patients. A disadvantage of the current SCEM design when compared to lead skin collimation is that there is the potential for collisions with the patient or immobilization equipment. Additionally, lead skin collimation allows the physician to directly place the lead on the area they want to shield and allows for better visualization of the area that will be treated. When compared to custom‐printed bolus, the SCEM can save time by eliminating the need to 3D print a bolus for individual patients. The current design of the SCEM, however, does not allow for range modulation as this could only be achieved with varying electron energies.

As previously discussed, there have been several studies on the use of photon multileaf collimators (MLCs).^8^, ^9^, ^10^, ^11^ While the use of photon MLCs for electron treatments allows electron fields to be more easily combined with photon fields (thereby eliminating the need for treatment interruption), there are several disadvantages. Plessis et al. demonstrated that beyond 70 cm SSD, the photon MLCs were not advantageous, as lower energy fields deviated from the project fields at this distance.^8^ Their study also showed the bremsstrahlung contamination to be comparable to conventional Cerrobend cutouts, thereby providing little to no benefit in the reduction of out‐of‐field dose and photon contamination. Mueller et al. calculated the dose for plans with SSDs varying from 70 to 100 cm and noted the potential for collisional issues at smaller SSDs and higher doses to nearby organs at risk (OAR) when extended SSDs were used.^10^ While the SCEM provides several advantages, using photon MLCs for electron therapy allows electron fields to be more easily combined with photon fields and does require treatment to be interrupted.^11^ Photon MLCs have been shown to have bremsstrahlung contamination comparable to conventional Cerrobend cutouts, thereby providing little to no benefit in the reduction of out‐of‐field dose and photon contamination.^8^ Because the SCEM is made of acrylic, the bremsstrahlung production is significantly reduced, thereby reducing the out‐of‐field dose. Additionally, to avoid collisions with a patient, photon MLCs are limited in how close they can be to a patient's surface. This leads to increased doses to organs at risk near the field.^10^ The SCEM concept allows for collimation closer to the patient's skin surface, thus reducing the penumbra and dose to areas outside the tumor volume.

Several groups have proposed designs for add‐on electron MLCs.^12^, ^13^, ^14^, ^15^ Lee et al. demonstrated that using photon MLCs for electron therapy was a poor choice for generating complex MLC shapes due to in‐air scattering and proposed an add‐on tungsten MLC at a source‐to‐collimator distance (SCD) comparable to traditional electron applicators. Despite the improvements in penumbra over photon MLCs, current add‐on electron MLCs still suffer from larger penumbra than with surface collimation, particularly for lower energies. O'Shea et al. showed some improvements in penumbra by using an extendable EMLC system (at 95 cm SCD), but even this design still had difficulty matching low‐energy fields.^15^ Several of these systems have also shown increased bremsstrahlung production due to the leaf material .^12^, ^13^, ^14^ While photon MLCs have been shown to increase in‐air scattering, an add‐on MLC system could allow for the field to be shaped closer to the patient's surface. Previous designs of add‐on MLC systems have used source‐to‐collimator distances (SCDs) comparable to traditional electron applicators.^12^, ^15^ These designs showed some advantages over photon MLCs but they did not show significant improvement in reducing the penumbra when compared to applicators with Cerrobend inserts, particularly for low energies. The SCEM provides decreased penumbra due to it's ability to collimate almost directly on the surface.

This work focused on comparing the current SCEM design with traditionally used Cerrobend cutouts. For all field sizes and energies, the d_max_ for the SCEM shifted towards the surface, and this effect was more pronounced with increasing energy. The surface dose relative to d_max_ for the SCEM was higher than for the Cerrobend cutout for all energies and field sizes, with the largest difference seen at 9 MeV for all three field sizes. The bremsstrahlung tail for the SCEM was lower than Cerrobend for all energies and field sizes. The SCEM showed a significant decrease in penumbra for all energies and field sizes when compared to the Cerrobend cutout at 110 cm SSD, particularly at shallower depths and lower energies. The largest improvement in penumbra for the 10 cm × 9 cm field was at 6 MeV, decreasing by 58.8% from the Cerrobend cutout at 110 cm SSD. The out‐of‐field dose for all field sizes and energies decreased when the SCEM was compared to the Cerrobend cutouts (for both 100 cm SSD and 110 cm SSD), with the largest decrease occurring for the 6 MeV 10 cm × 9 cm field.

Changes in the output factor are affected by the establishment of lateral equilibrium and also scatter from outside the phantom. The two setups, one with solid water and one with the MiniPhantom, were used to evaluate output changes. The MiniPhantom has a fixed width of 3 cm which allows for an evaluation of output changes without increased lateral scatter with field size. In the MiniPhantom above 6 MeV, the outputs for the Cerrobend cutouts are relatively stable. However, the outputs for the SCEM vary by as much as 7% (for the 5 cm × 9 cm field at 15 MeV) when compared to the reference field size of 10 cm × 9 cm. Since there is no change in the amount of phantom being irradiated, the large differences in output must be due to the additional scatter off of the SCEM surface. The detector and phantom setups were chosen to avoid any biasing of one field size over another. Because of the larger surface area of the SCEM leaves (compared to the Cerrobend cutouts), the output factors for the smaller field sizes are higher than for the 10 cm × 9 cm. The larger scattering surface being closer to the detector allows for higher output. We believe that the 3 cm × 9 cm output is less than the 5 cm × 9 cm output due to the fact that the scattering angle for electrons reaching the detector is more restricted for a smaller field size.

**FIGURE 11:**
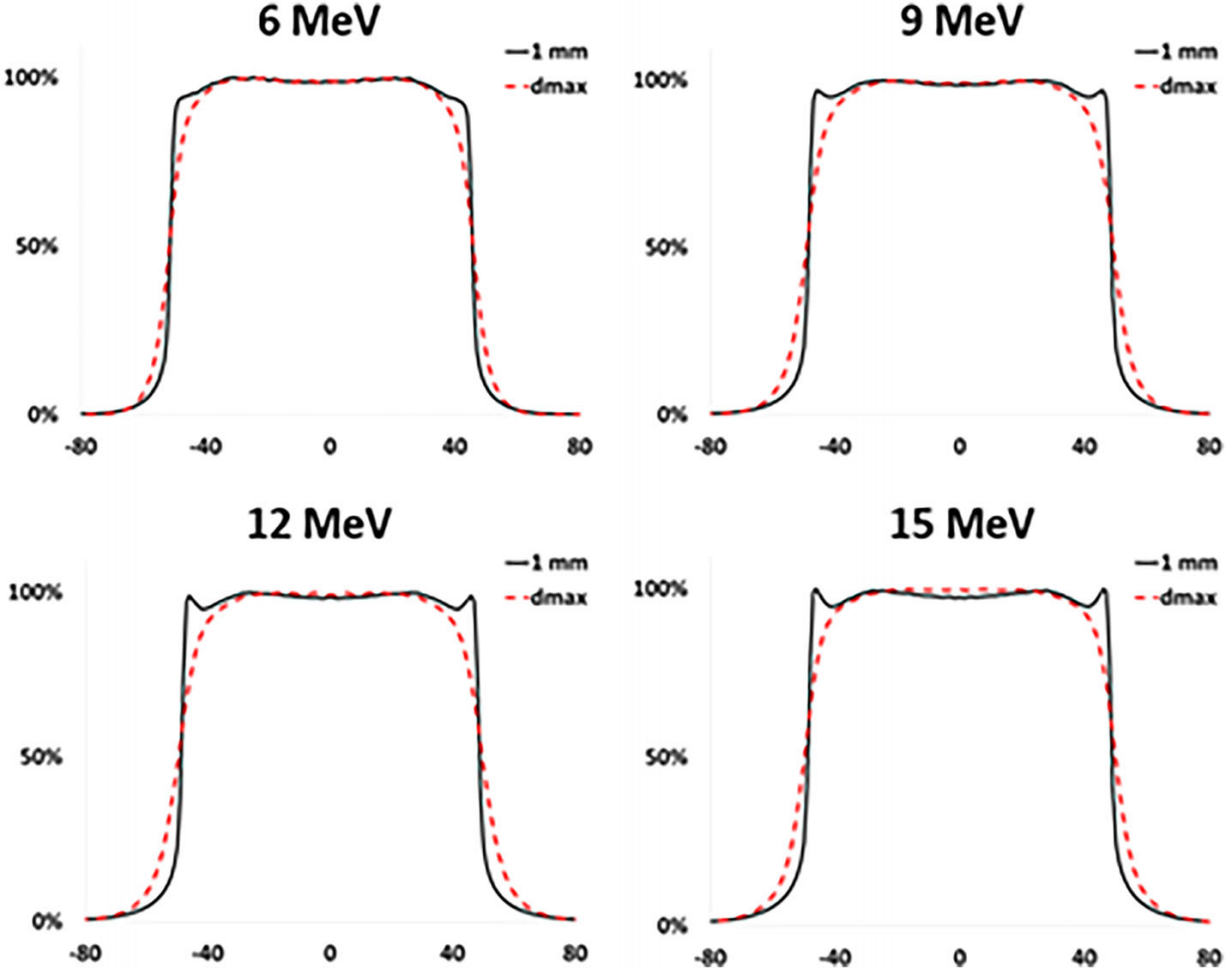
Cross‐plane profiles for the SCEM for the 10 cm × 9 cm field size at 1 mm depth (near‐surface) and d_max_ for each energy.

A disadvantage of the SCEM is the “horns” on the shallow depth field edges for higher energies at the largest field size, as seen in Figure 11. While this effect diminishes with increasing depth, it provides sub‐optimal dosimetry near the surface. Higher skin dose increases the risk for skin reactions, cosmetic effects, and other undesirable side effects. A similar effect was seen by Rusk et al. when they compared copper cutouts with Cerrobend cutouts.^23^ The “horns” on the field edges are likely to due the larger scattering surface of the SCEM when compared to the Cerrobend cutouts. This effect should decrease with increasing the atomic number of the collimating material (i.e., decreasing the thickness needed to collimate the electron beam decreases the scattering surface). Future work will be dedicated to determining a denser material that can decrease the scattering surface size of the leaves without increasing the among of Bremsstrahlung produced. In addition to the shallow depth “horns”, the current design of the SCEM is large and bulky. This is primarily because the leaves are made of acrylic and require a greater thickness to shield higher energy electrons. Using a higher Z material could help to reduce the overall size of the device.

Despite the abovementioned disadvantages, the SCEM provides several advantages over Cerrobend cutouts. In addition to being toxic and posing various health risks, Cerrobend cutouts must be poured for each field to be treated. The results of this work have helped to characterize the dosimetric parameters of the existing device and have helped establish areas of improvement for the SCEM.

## 5 CONCLUSIONS

This work demonstrates an early concept for an add‐on SCEM. A prototype consisting of acrylic MLCs that protrude towards the patient surface was designed to allow for more conformal electron radiotherapy treatments that would reduce the penumbra as well as the Bremsstrahlung production. Water tank scans including PDD and profile measurements were made for the SCEM and for Cerrobend cutouts of various, matched field sizes. Additionally, output factor measurements using solid water and the MiniPhantom were made for both the SCEM and Cerrobend cutouts. The objective was to characterize the dosimetric properties of an early prototype to determine how it could be improved upon. The initial design provides several benefits over the current standard of applicators with custom‐made Cerrobend inserts. When compared to Cerrobend cutouts, the SCEM reduced penumbra by up to 58.8%. Additionally, their construction of a low‐Z material lowers the bremsstrahlung production significantly, thereby decreasing the out‐of‐field dose. The SCEM reduced the out‐of‐field dose by up to 92.3%. Future work should include improving sub‐optimal dosimetric characteristics of the SCEM, particularly the “horns” seen in shallow‐depth profiles at high energies for larger field sizes and the increase in shallow‐depth PDDs. This may necessitate the use of a different material for the leaves. We plan to develop a method for controlling leaf motion in both tangential and normal directions. Additionally, we will investigate the use of the SCEM in MERT. While this study has helped to identify areas of improvement for the SCEM, the initial prototype of the SCEM has shown promising result that can have a meaningful impact on electron radiotherapy, particularly in the context of out‐of‐field dose reduction through improved penumbra and decreased Bremsstrahlung production.

## AUTHOR CONTRIBUTIONS

All authors contributed to the design of the work, interpretation of the data, and preparation of the manuscript.

## CONFLICT OF INTEREST STATEMENT

Two of the authors hold a patent on the technology described in the present study.
